# *SERPINB11* Frameshift Variant Associated with Novel Hoof Specific Phenotype in Connemara Ponies

**DOI:** 10.1371/journal.pgen.1005122

**Published:** 2015-04-13

**Authors:** Carrie J. Finno, Carlynn Stevens, Amy Young, Verena Affolter, Nikhil A. Joshi, Sheila Ramsay, Danika L. Bannasch

**Affiliations:** 1 Department of Population Health and Reproduction, School of Veterinary Medicine, University of California Davis, Davis, California, United States of America; 2 Department of Animal Science, University of California Davis, Davis, California, United States of America; 3 Department of Pathology, Microbiology and Immunology, School of Veterinary Medicine, University of California Davis, Davis, California, United States of America; 4 Genome Center Bioinformatics Core, University of California Davis, Davis, California United States of America; 5 Institute of Veterinary, Animal and Biomedical Sciences (IVABS) Massey University, Palmerston North, New Zealand; Stanford University School of Medicine, United States of America

## Abstract

Horses belong to the order Perissodactyla and bear the majority of their weight on their third toe; therefore, tremendous force is applied to each hoof. An inherited disease characterized by a phenotype restricted to the dorsal hoof wall was identified in the Connemara pony. Hoof wall separation disease (HWSD) manifests clinically as separation of the dorsal hoof wall along the weight-bearing surface of the hoof during the first year of life. Parents of affected ponies appeared clinically normal, suggesting an autosomal recessive mode of inheritance. A case-control allelic genome wide association analysis was performed (n_cases_ = 15, n_controls_ = 24). Population stratification (λ = 1.48) was successfully improved by removing outliers (n_controls_ = 7) identified on a multidimensional scaling plot. A genome-wide significant association was detected on chromosome 8 (p_raw_ = 1.37x10^-10^, p_genome_ = 1.92x10^-5^). A homozygous region identified in affected ponies spanned from 79,936,024-81,676,900 bp and contained a family of 13 annotated *SERPINB* genes. Whole genome next-generation sequencing at 6x coverage of two cases and two controls revealed 9,758 SNVs and 1,230 indels within the ~1.7-Mb haplotype, of which 17 and 5, respectively, segregated with the disease and were located within or adjacent to genes. Additional genotyping of these 22 putative functional variants in 369 Connemara ponies (n_cases_ = 23, n_controls_ = 346) and 169 horses of other breeds revealed segregation of three putative variants adjacent or within four *SERPIN* genes. Two of the variants were non-coding and one was an insertion within *SERPINB11* that introduced a frameshift resulting in a premature stop codon. Evaluation of mRNA levels at the proximal hoof capsule (n_cases_ = 4, n_controls_ = 4) revealed that *SERPINB11* expression was significantly reduced in affected ponies (p<0.001). Carrier frequency was estimated at 14.8%. This study describes the first genetic variant associated with a hoof wall specific phenotype and suggests a role of *SERPINB11* in maintaining hoof wall structure.

## Author Summary

Inherited diseases affecting only the nails in humans are rare; however, humans do not support themselves entirely on one appendage. Horses bear their entire weight on their third toe, resulting in a large amount of force on each hoof. An inherited disease characterized by a phenotype restricted to separation and breaking of the dorsal hoof wall was identified in a specific breed of pony, the Connemara. This disease has been termed hoof wall separation disease (HWSD). Parents of affected ponies appeared clinically normal, suggesting an autosomal recessive mode of inheritance. A genome-wide association analysis identified a region associated with HWSD which was further assessed through whole genome next-generation sequencing and genotyping of potential variants. Here, we present the discovery of a frameshift variant, leading to a premature stop codon in *SERPINB11* of HWSD-affected ponies. Significantly decreased expression of the *SERPINB11* transcript was identified in the hoof capsule of HWSD-affected ponies. This study describes the first genetic variant associated with a hoof wall specific phenotype and suggests a role of *SERPINB11* in maintaining hoof wall structure.

## Introduction

Many of the signaling molecules involved in patterning ectodermal derivatives, such as teeth and hair, are also involved in organizing mammalian distal limb appendages, including nails, claws and hooves [1]. Perissodactyla (odd-toed ungulates) are a diverse group of mammals that include the horse, zebra and donkey. These animals bear their weight almost entirely on the third toe. In the modern horse, the non-weight bearing digits have atrophied while the third digit has enlarged. The nail has also evolved to become fully interdigitated with the underlying soft tissue and to form a full weight bearing structure, the hoof. As horses are prey animals, the development of hooves illustrates a major evolutionary innovation based on the need for rapid acceleration and sustained speed. Since only the hooves touch the ground, the remaining portions of the foot have become parts of the limb, substantially increasing the length of stride. Additionally, raising the heel and digits off the ground increased the number of joints that move the limbs forward and thereby increased the rate of stride. Although these modifications substantially increase the potential speed and acceleration of these animals, extensive structural integrity of the hoof is required to support all of the body weight. On average, adult horses and ponies weigh 1000 and 880 lbs, respectively. At an evenly balanced stance when motionless, this places an average of 220–250 lbs of force on each limb. When in motion, ground reaction forces increase at various phases of the gait, resulting in additional force applied to each hoof [2].

Ectodermal dysplasias (EDs) are a heterogenous group of congenital disorders characterized by alterations in two or more ectodermal structures (hair, teeth, nails or sweat glands) [3]. Genetic conditions that exclusively involve the nails are rarer and have been classified as nonsyndromic congenital nail disorders (NDNC). Currently, there are ten characterized NDNCs in people, of which four have associated genetic alterations. Leukonychia (NDNC3), characterized by white discoloration of the nails, is caused by an alteration in *PLCD1* [4]; Anonychia/hyponychia (NDNC4, absence of hypoplasia of nails) due to an alteration in *RSPO4* [5]; toenail dystrophy (NDNC8), caused by an alteration in *COL7A1* [6] and onychodystrophy (NDNC10), associated with an alteration in *FZD6* [7]. There are currently six NDNCs for which a genetic alteration has not been identified, including isolated congenital onychodysplasia (NDNC7), a disease characterized by longitudinal streaks, thinning and splitting at the distal nail edge of all finger and toenails [8].

This study describes an inherited disorder, termed Hoof Wall Separation Disease (HWSD), characterized by separation and breaking of the dorsal hoof wall in the Connemara pony. Without the integrity of the hoof wall, ponies cannot support their weight effectively. The associated chronic inflammation often leads to laminitis, a debilitating condition characterized by separation of the third phalanx from the epidermal laminae that connect the bone to the dorsal hoof wall. Chronic laminitic episodes in horses are very painful and often warrant euthanasia. There are no known alterations that affect the hoof wall in horses and a comparative approach was not feasible due to the unique nature of the hoof. Our goal was to identify the molecular etiology of the disease in order to reduce its prevalence through genetic testing and to provide insight into this unique ectodermal structure. Genome-wide association analysis, coupled with whole genome next-generation sequencing, identified a frameshift variant in *SERPINB11* associated with this novel, hoof specific phenotype in Connemara ponies. SERPINB11 remains an uncharacterized protein in humans [9,10] and further investigation of the potential role of SERPINB11 in NDNCs may be warranted.

## Results

### Phenotypic Description

**Fig 1:**
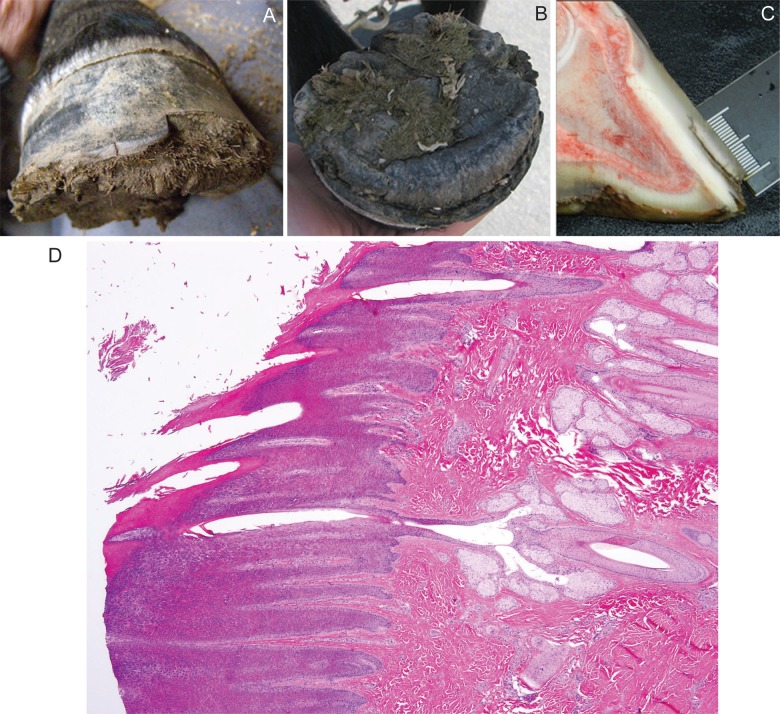
Hoof from a Connemara pony foal affected with hoof wall separation disease (HWSD). (A) Note the dorsal hoof wall separation at the sole and (1B) proliferative horn on the solar aspect of the hoof (1B). (1C) A sagittal section of a post-mortem HWSD-affected hoof demonstrates that the dorsal hoof wall fissure occurs outside of the white line. (1D) Coronary band, periople (hematoxylin & eosine stain): normal transition from haired skin into periople is evident.

Two clinically-affected Connemara females were examined at 5-months and 1-year of age. The onset of hoof pathology in these two index cases had become evident at 3 and 5-months of age, respectively. With the exception of the hooves, physical examinations revealed no other abnormalities; haircoat, underlying skin, mucous membranes and mucocutaneous junctions appearing normal. Abnormal sweating was not reported in either case. In both cases, all four hooves displayed a dorsal hoof wall separation at the sole with a normal coronary band appearance (Fig 1A). Proliferative horn was evident on the solar aspect of all four hooves (Fig 1B). The 5-month old pony, which had markedly proliferative solar horn, was lame on both front feet at the walk while the yearling, which had undergone a recent hoof trimming, appeared sound at the walk. Distal extremity radiographs of both front feet and the dental arcade revealed no abnormalities.

Hooves from three additional HWSD-affected female ponies (aged 1, 4 and 5 years) underwent complete gross examination. Age of onset in these three cases was less than 6-months of age. In the three cases, all four hooves showed variable degrees of splitting within the dorsal hoof wall, most prominent along the distal margin and variably spreading more proximally. The horn of the hoof wall was brittle and easily broken while the horn of the sole appeared stronger. The coronary band appeared normal. All four feet were bisected sagittally. Coffin bone rotation, an indication of laminitis where the toe of the distal phalanx (i.e. coffin bone) has dropped due to loss of lamellae support, was evident in 2/3 ponies (aged 4 and 5 years). The white line was intact and there was no hyperaemia or scarring in the corium or lamina. In the one HWSD pony (1-year of age) with no evidence of coffin bone rotation, the distance of the white line to the horn capsule measured 1 cm and was consistent proximal to distal. This horn to white line distance is within the normal radiographic reference range reported for adult ponies [11]. The dorsal hoof wall separation was outside of the white line (Fig 1C). Histologic examination of coronary band (Fig 1D), periople and proximal lamina, skeletal muscle and liver performed in one of three ponies (1-year of age) revealed no pathologic changes.

### Genome Wide Association (GWA)

**Fig 2:**
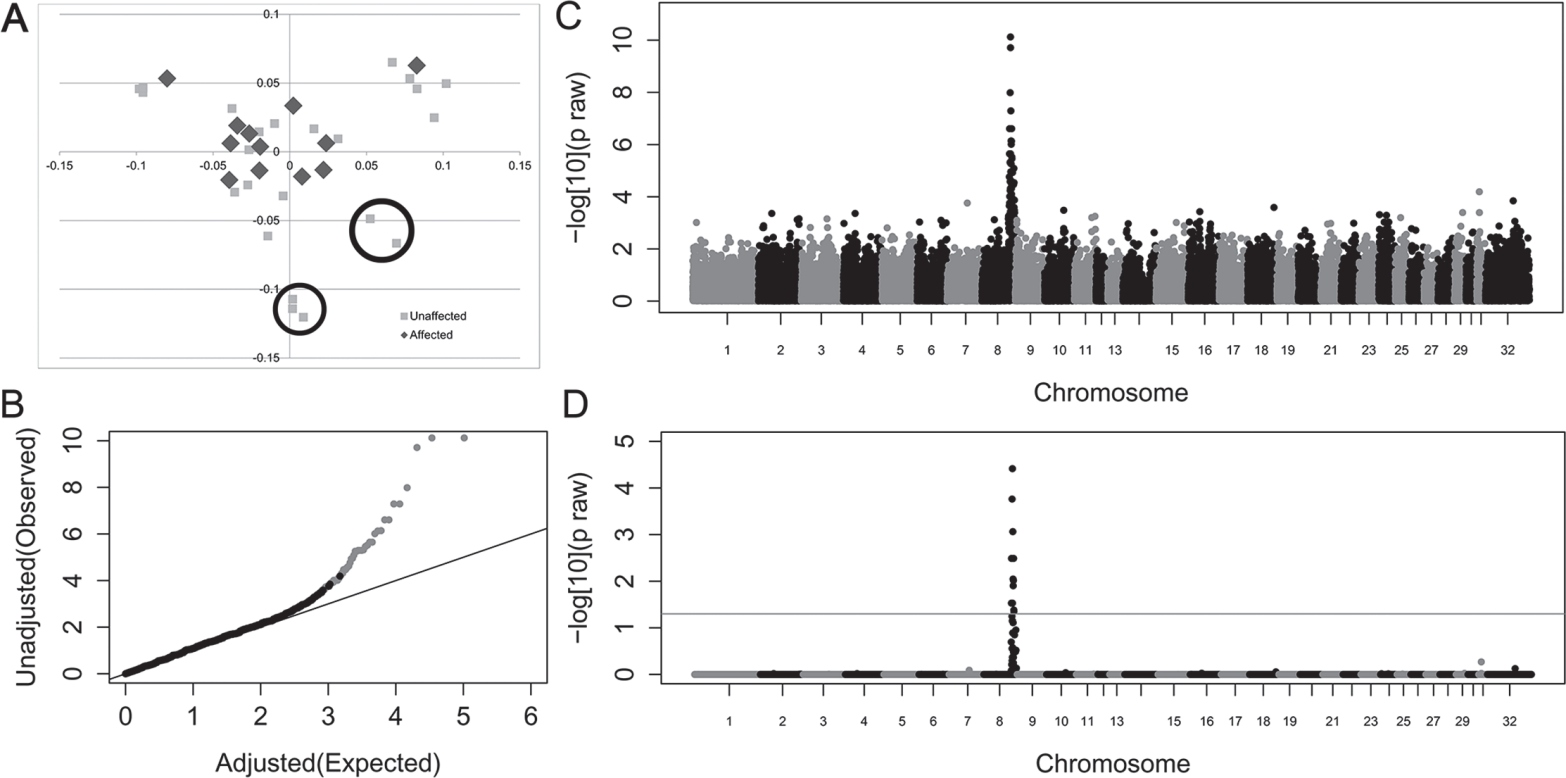
Genome-wide case-control allelic association study results of Connemara ponies with HWSD (15 cases, 24 controls). (2A) Multi-dimensional scaling plot. Controls circled contributed most to genomic inflation and were removed from analysis (2B). Quantile-Quantile plot of genome-wide association results after removing 7 control ponies. Grey dots represent the observed versus expected p-values of all SNPs. Black dots represent the observed versus expected p-values after removal of all SNPs on chromosome 8. The solid grey line represents the null hypothesis: observed p-values equal expected p-values. (2C) Manhattan plot of—log_10_ of raw p-values by chromosome. The lowest p-values are on chromosome 8. (2D) 52K Max (T) permutation results. The line that parallels the X axis denotes genome-wide significance (p<0.05;-log_10_ >1.3).

A case-control allelic GWA was performed on the 51,453 SNPs that passed quality control. Initial genomic inflation (λ = 1.48) was reduced (λ = 1.09) by eliminating seven unaffected outliers identified by multi-dimensional scaling (Fig 2A and 2B). Within this new sample set, there was a positive association between HWSD and a 1.7 Mb region on equine chromosome 8 (Fig 2C and 2D (S3 Table)); top SNVs at chr8: 80,772,490 and chr8:80,648,576 p_raw_ = 1.37x10 ^= 10^, p_corrected_ = 1.92x10^-5^). Ponies affected with HWSD had a distinct homozygous haplotype within the identified region; the same haplotype was not observed in control animals in the homozygous state. SNP genotypes of the associated region are provided in S4 Table.

### Variant Identification

**Fig 3:**
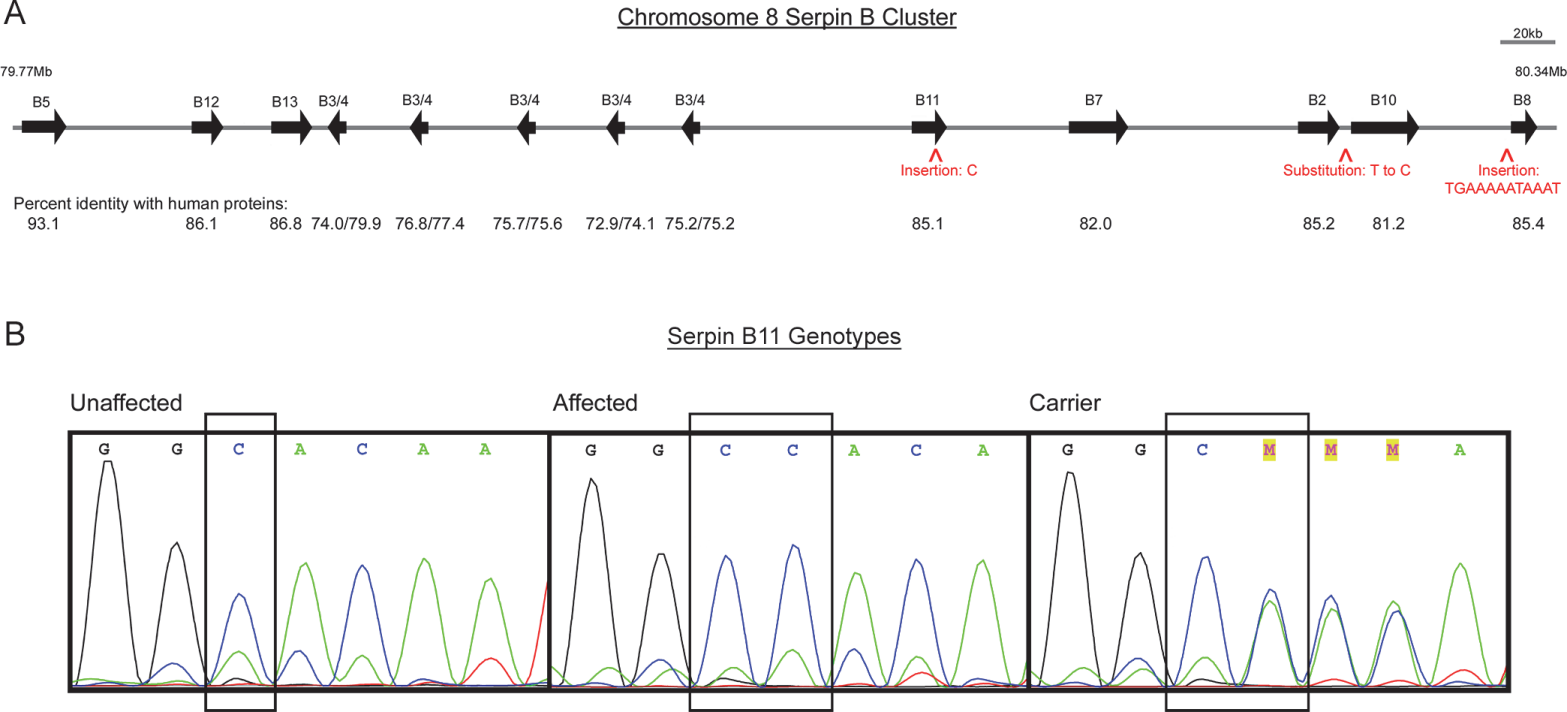
Genes and segregating variants within the region of homozygosity shared by HWSD-affected ponies. (3A) Scaled schematic representation of *SERPINB* gene family situated between 79.77Mb and 80.34Mb on equine chromosome 8. Arrows show the relative positions of the *SERPINB* genes in the cluster, and aligned numbers report the percent identity of the horse proteins compared to corresponding human proteins at the amino acid level. Five copies of equine *SERPINB3/4* are variably closer to human B3 or B4. Carets depict locations of variants that segregate with HWSD: two insertions and one substitution. (3B) Chromatograms of *SERPINB11* exon 5 sequences where a variant of C to C/C was found in affected Connemara ponies. From left to right: unaffected ponies have a genotype of “C/C”; affected Connemara ponies have a genotype of “CC/CC”; carrier Connemara ponies have a genotype of “C/CC”.

The homozygous region identified in affected ponies (79,936,024–81,676,900 bp), contains a family of *SERPINB* genes. Based on the *Equus caballus* 2.0 genome assembly [12], horses have three additional copies of *SERPINB3/B4* within this interval as compared to humans (Fig 3A). The rest of the genes, orientations and order are conserved with respect to human. Due to limited information regarding comparative diseases related to the *SERPIN* gene family, whole-genome next generation sequencing was performed instead of selectively sequencing predicted candidate genes. Whole-genome sequencing was performed on two Connemara HWSD cases and two unaffected Connemara ponies that were homozygous reference for the associated haplotype. A published Quarter horse whole-genome sequence was used as an additional control [13].

Sequencing revealed a total of 9,758 single nucleotide variants (SNVs) within the interval, of which 363 segregated with HWSD in the two cases and three controls (S5 Table). Of these 363 SNVs, 16 were located within annotated genes or were fewer than 700 base pairs from the ATG. Although the promoters for the genes are not identified in horses, we selected 700 base pairs in order to cover key regulatory regions close to the start of translation. One additional SNV was chosen based on its location between SerpinB2 and SerpinB10. These gene-associated SNVs were selected for follow-up genotyping within a larger sample set. In addition, sequencing identified 1,230 small insertions and deletions (indels), of which 28 segregated with disease (S6 Table); 11 were within or close (<700 bp) to genes. Of these 11 indels, 6 were intronic, 1 was coding, and 4 were up/downstream. The coding and up/downstream indels were tagged for genotyping in a larger sample set. Of the 22 variants (17 SNVs and 5 indels) genotyped on the custom Sequenom panel, 21 passed quality control. Three variants were unique to the 23 affected Connemara ponies and heterozygous in 27 obligate carriers (Table 1). Although one indel failed Sequenom genotyping, we retained it in our analysis (Table 1). None of these variants were identified in the 169 non-Connemara equines also genotyped on the Sequenom array, however 82 unaffected Connemara ponies were heterozygous for the three variants and 244 were wild-type.

**Table 1:** Variants segregating with HWSD phenotype.

**Genomic Location**	**Variant Type**	**Effect**	**gDNA**	**cDNA**	**Protein**
chr8:80111598	1 bp-insertion	Frameshift *SERPINB11*	g.80111598_80111599insC or:g8883_8884insC	c.504_505insC	p.Thr169Hisfs*3
chr8:80259666	SNV	Downstream; 1431bp from *SERPINB2* and upstream; 2596 bp from *SerpinB10*	g.80259666T>C	N/A	N/A
chr8:80319671	12 bp-insertion	Upstream; 691bp from *SerpinB8*	g.80319671_80319683insTGAAAAATAAAT	N/A	N/A
chr8:80319673	4-bp deletion	Upstream; 677bp from *SerpinB8*	g. 80319673del	N/A	N/A

Four variants were unique to the 23 affected Connemara ponies and heterozygous in 27 obligate carriers.

On chromosome 8, base pair 80,259,666 is located downstream of *SERPINB2* and upstream of *SERPINB10*; 80,319,671 and the position of the four-base-pair deletion that failed quality control (80,319,673) are both within the rolling circle (RC) repeat element Helitron3Na_Mam located upstream of *SERPINB8*; 80,111,598 is in the fifth exon of *SERPINB11* (Fig 3). The insertion within *SERPINB11* introduces a frameshift that first alters the two amino acids following residue 168, and then introduces a premature STOP codon. 55% of the protein is predicted to be truncated, including a large portion of the serpin scaffold and the entire reactive site loop [9].

### Gene Expression

**Fig 4:**
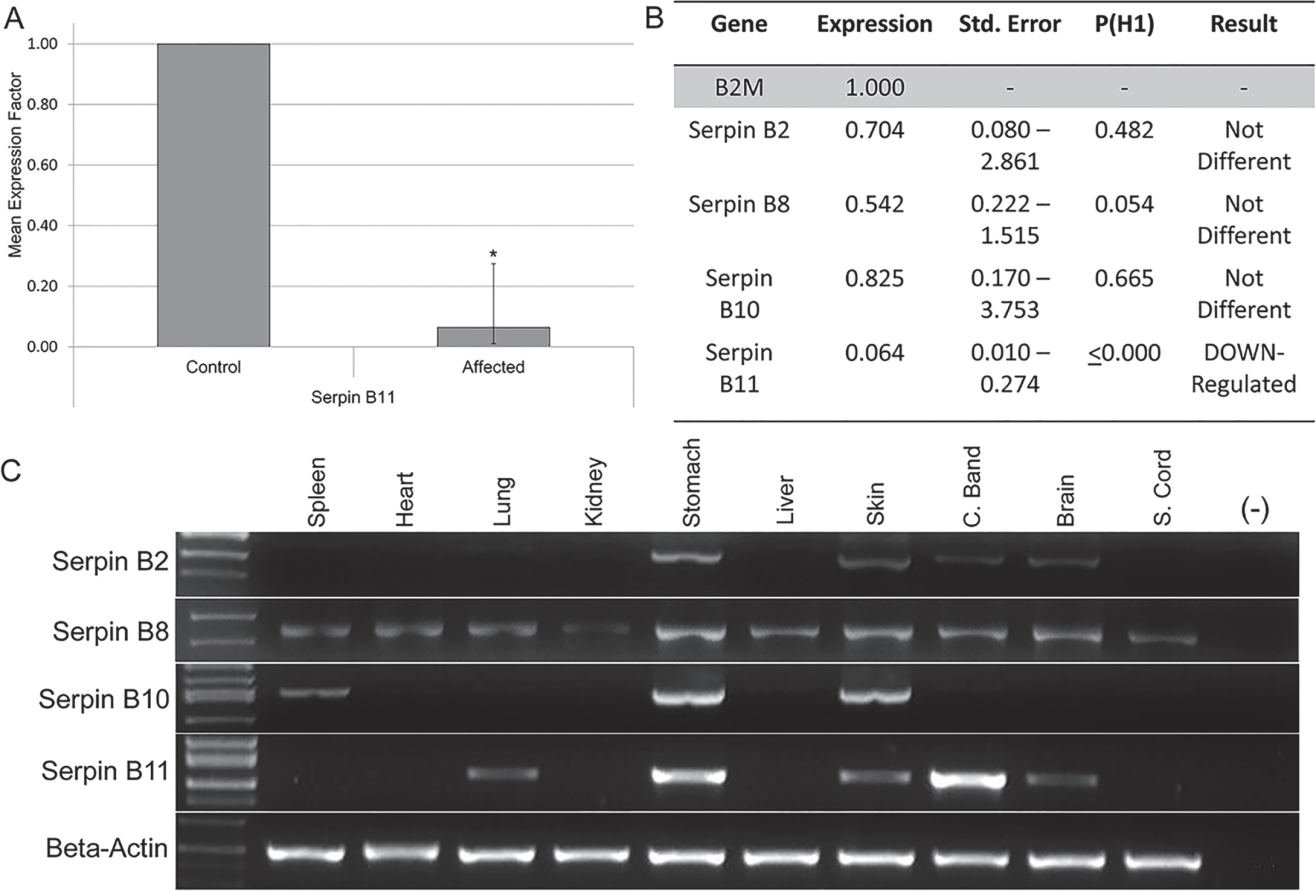
Expression analysis of *SERPINB2*, *SERPINB8*, *SERPINB10* and *SERPINB11*. (4A) Relative *SERPINB11* gene expression ratio of coronary band cDNA from four HWSD-affected Connemara ponies compared to four unaffected horses. Relative expression levels were normalized to the housekeeping gene B2M. Statistical significance is reported as p<0.001(*). B. Relative *SERPINB2*, *B8*, *B10*, *and B11* gene expression levels in four affected Connemara ponies, compared to four unaffected horses and normalized to the housekeeping gene *B2M*. P(H1) = Probability of alternate hypothesis that difference between sample and control groups is due only to chance. P(H1) is significant (p<0.05) for only *SERPINB11*. C. Gel image of PCR amplification of *SERPINB2*, *B8*, *B10*, *and B11* from spleen, heart, lung, kidney, stomach, liver, skin, coronary band, and spinal cord cDNA of an unaffected horse. Beta-actin was used to control for cDNA concentrations.

As potential regulatory variants were not identified in our analysis and in order to prioritize the four remaining candidate variants, expression analysis of *SERPINB2*, *SERPINB8*, *SERPINB10 and SERPINB11* was performed. These were the four genes closest to segregating SNVs and indels (Fig 3). Evaluation of mRNA levels in coronary band samples from four HWSD-affected ponies and four unaffected controls revealed that coronary band *SERPINB11* expression was significantly reduced in affected ponies. Relative Expression Software Tool (REST) analysis indicated down-regulation (0.064; S.E. range 0.010–0.274) in the affected group by a mean factor of 16 and a probability that the difference between sample and control groups was due only to chance [P(H1)] of <0.001 (Fig 4A). By contrast, REST showed no difference in expression of *SERPINB2*, *SERPINB8*, or *SERPINB10* between the affected and unaffected sample groups (Fig 4B).

*SERPINB11* was also found to be a very abundant transcript in the coronary band of an unaffected horse, relative to its levels in other tissues. RT-PCR showed gene expression in lung, stomach, skin, coronary band, and brain. Levels were subjectively highest in the stomach and coronary band. By contrast, *SERPINB2* appeared most highly expressed in stomach and skin; *SERPINB8* was widely expressed and most abundant in stomach and skin; *SERPINB10* was minimally expressed in coronary band and most prominent in stomach and skin (Fig 4C).

### Penetrance, Allele Frequency, and Carrier Frequency

The *SERPINB11* frameshift variant was found to be homozygous in a total of 31 affected ponies indicating complete penetrance. The severity of phenotype ranged from mild (cleft between dorsal hoof wall and white line apparent on solar aspect of hoof but the pony was able to be maintained with frequent hoof trimming and shoeing) to severe (Fig 1). Within the entire 423-Connemara-pony data set, allele frequency was 18.7% and a total of 96 ponies were heterozygous for the *SERPINB11* insertion. The heterozygous animals are all phenotypically unaffected by HWSD. Within a 324-pony subset of individuals unrelated to the affected animals, carrier frequency for the variant is 14.8%.

## Discussion

### Phenotype

Based on our clinical and histologic assessment, we have defined the phenotype of HWSD in the Connemara pony to include an early age of onset (within the first 6-months of life) and characteristic splitting and separation of the dorsal hoof wall (Fig 1A). Lesions are specific to the dorsal hoof wall and do not appear to involve any other ectodermal structures. Laminitis may be a sequelae to HWSD.

Splitting of the dorsal hoof wall may be observed in inflammatory or infection conditions of the equine hoof, including white line disease or as a complication of laminitis or solar abscessation. For this study, we characterized HWSD-affected ponies based upon the following criteria: (1) Connemara pony breed (2) age of onset within the first six months of life and (3) characteristic clinical signs, supported by digital photographs of all four feet. Other diseases, including white line disease and abscessation, are associated with infection; the sole or white line is visibly discolored and unhealthy on examination. With HWSD, the sole and white line appear healthy, with the exception of proliferative sole in the animal’s effort to support the limb on a structure other than the dorsal hoof wall. The Connemara pony originated in the County Galway of western Ireland and the breed standard is characterized by hard strong hooves [14]. These ponies are often unshod when used for performance activities (jumping, dressage, driving), evidence of a strong and healthy hoof within the breed. Based on the breed standard, this makes the phenotype of HWSD all the more striking in affected individuals. The age for the control ponies within this study was set at >2 years. We have not observed any reports of an older age of onset of HWSD; most ponies are affected within the first 6 months of life.

In the 23 ponies positive for the identified *SERPINB11* variant, all demonstrated an abnormal dorsal hoof wall. However, variable expressivity was apparent in HWSD-affected cases. Mild HWSD cases demonstrated evidence of the dorsal hoof wall separation on the solar aspect of the hoof without severe splitting evident on a lateral view. In more severely affected cases (Fig 1), the dorsal hoof wall separation was readily apparent. We did not identify any potential genetic modifiers that could account for the variability in phenotype. Owners of HWSD-affected ponies often notice that the splitting of the distal hoof wall worsens when the environmental conditions change from dry to wet or vice versa. Management typically consists of frequent trimming of hooves and, in some cases, glue-on shoes as the dorsal hoof wall of most HWSD-affected ponies will split if nails are used.

The dorsal hoof wall is composed of keratins, which provide strength, hardness and insolubility due to disulfide bonds between and within the long chain fibrous molecules [15]. There are dozens of different keratin molecules, with molecular weights in the range of 40–70 kDa and varying degrees of hardness and sulfur concentration [16]. Terminally differentiated keratinocytes, originating from the coronary band, are arranged in specialized tubular and intertubular configurations in four distinct zones [17]. The gradient in tubule density mirrors the gradient in water content across the hoof wall, with the innermost layers of the hoof having the highest relative water content, which confers high crack resistance [18]. In healthy horses, by the time the shock of the impact with the ground reaches the first phalanx, about 90% of the energy has been dissipated, mainly at the innermost hoof wall layer (i.e. the lamellar interface) [19]. Distal hoof wall splitting does not, in it of itself, result in lameness. Rather, repeated stress on the innermost layer of the lamellar interface results in separation of the interdigitating lamellae from the distal phalanx (i.e. laminitis). As laminitis progresses, radiographs of the distal phalanx may reveal the separation of the dorsal hoof wall from the distal phalanx. Although radiographs were unremarkable in the examined 5-month old filly, the lameness was most likely due to lamellar inflammation that had not yet progressed to full separation.

### Genome-Wide Association

The original population of ponies used in this study was stratified, which was markedly improved by removing seven control ponies visualized as outliers on the multidimensional scaling plot (Fig 2A). Alternatively, a mixed model approach could have been utilized to correct for the level of stratification; however, removal of the seven control ponies did not affect power to detect a significant association on ECA8. The associated region on ECA8 encompassed ~1.7 Mb, which contained 13 annotated *SERPBINB* genes. None of these genes have been previously associated with any of the human EDs nor was there any supporting evidence documenting expression of these genes in any ectodermal structures.

### Whole Genome Sequencing Reveals a Frameshift Variant in *SERPINB11*

Targeted re-sequencing of the ~1.7 Mb candidate region was considered; however, it has become more cost effective to perform whole-genome sequencing, when an autosomal recessive mode of inheritance is suspected. With Mendelian disorders, putative functional variants may more readily be uncovered on smaller sample sets using whole genome sequencing whereas if a more complex mode of inheritance was likely, capture re-sequencing could allow for many individuals to be sequenced for the particular region of interest at a relatively comparable cost [20].

Sequencing revealed 363 SNVs and 28 indels that segregated with HWSD, with 17 and 11, respectively, located within or adjacent to annotated genes. After genotyping a larger population of HWSD-affected and unaffected equids, four segregating variants remained (Table 1), including a 4-bp deletion that had failed Sequenom genotyping. Of these four variants, only one was coding. The insertion within *SERPINB11* introduced a frameshift, leading to a premature stop codon. Based upon the severity of the HWSD phenotype, priority was placed on coding variants as we presumed the variant would alter the amino acid structure of the protein involved. In addition, q-RT-PCR data demonstrated decreased expression of *SERPINB11* in coronary band tissue, where keratinocytes of dorsal hoof wall originate. The decision to focus on the one coding variant for HWSD was validated in this study; however, we acknowledge that non-coding variants have been increasingly associated with disease [21]. The three other HWSD-segregating variants may be a part of the haplotype on which the frameshift variant originated.

### *SERPINB11* in Humans

Across species, serpins represent that largest and most functionally diverse family of serine protease inhibitors. Some serpins exhibit alternative functions, such as hormone transport and blood pressure regulation [22]. Serpins have been classified into clades according to their sequence similarity. Clades are classified as A-P, with clades A-I representing human serpins [22]. Serpins have a highly conserved secondary structure, with three β-sheets (A, B and C), nine α-helices and a reactive center loop (RCL), which serve as bait for the target proteases. The tertiary structure allows for a conformational change critical to protease inhibitor activity [22]. Serpins exist as monomeric proteins in their native state, which is defined by an exposed RCL that allows it to interact with the protease. Serpins can transition back and forth between latent and active forms [10].

The SERPINB clade is considered the ovalbumin or ov-serpin clade, based on their high sequence similarity to chicken ovalbumin, and exist intracellularly [23]. In humans, clusters of genes on HSA6 and 18 have evolved from a common ancestor by one or two interchromosomal duplications with several intrachromosomal duplications [24]. A similar clustering is evident in the horse, with the clusters of *SERPINB* genes located on ECA8 and 20 [12]. Of the clade B serpins, only human SERPINB11 and mouse Serpinb11 have yet to be characterized [9,10]. Current evidence suggests that, in mice, *Serpinb11* can function as a trypsin inhibitor yet *SERPINB11* has lost inhibitory activity in humans and may have evolved a non-inhibitory function [9]. The inhibitor function of SERPINB11 has not been assessed in the horse. In the chicken, there is no orthologue for human SERPINB11, suggesting that these genes were either lost in the chicken or arose after the merging of avian and mammalian lineages [25]. Based on these limited studies of SERPINB11, it may be that there exist different roles, including varying abilities to function as protease inhibitors, of SERPINB11 between species.

Most research efforts into the characterization of major structural proteins of the equine hoof wall are targeted at the lamellae, as this is the site of biomechanical failure in equine laminitis. One study focused on characterizing proteins of the equine hoof wall that included the laminar epithelium (i.e. stratum internum), outer highly cornified hoof wall (stratrum medium) and coronary band epithelium [26]. From this investigation, it was evident that the keratin types within the coronary band epithelium were highly similar to those found in the stratum medium. Additionally, the high-sulfur and high-tyrosine protein components were rich in cysteine only in the stratum medium. Experimental studies from the same laboratory have determined that ^35^S-cysteine is preferentially taken up into the terminally differentiating hoof wall layers [26]. These cysteine-rich proteins are thought to contribute to stabilization of the interfibrillar matrix of the stratum medium through disulfide bonding. If SERPBINB11 retains cysteine peptidase inhibitory activity in the horse, as it does in the mouse [9], it may function to inhibit proteolytic cleavage of cysteine residues in the terminally differentiating hoof wall layer. A loss of SERPINB11 function could therefore result in a loss of peptidase inhibition and structural failure of the cysteine-rich hoof wall upon impact. Alternatively, SERPINB11 may play a specific role in relative keratinocyte proportions in the equine hoof wall, thereby weakening the overall structure and allowing it to fragment at the most distal end. Although keratinocytes may appear histologically intact at the level of the coronary band, the most distal aspect (i.e. most mature portion of the cell that is undergoing the majority of concussive impact) may be structurally abnormal. The distal dorsal hoof wall is difficult to evaluate histologically unless tissue is embedded in acrylic, which was not performed in this study. Further histologic investigation of the entire population of mature keratinocytes within the dorsal hoof wall of HWSD-affected ponies is warranted.

A bacterial artificial chromosome transgene expressing Cre under the control of *Serpinb7* regulatory elements was recently developed [27]. Although the original aim of the study was to evaluation the expression in kidney mesangial cells, the authors discovered that the *Serpinb7-Cre* transgene mediated *loxP*-recombination in all epidermal layers of the skin, hair follicle cells and the epithelium of the mouse forestomach and esophagus. The transgene colocalized with *Keratin10* and *Keratin14* in the suprabasal and basal layer of the epidermis, respectively [27]. Similar to these expression patterns, the expression of *SERPINB11* in our study was highest in the coronary band and stomach, with expression also detected in skin.

In humans, *SERBPINB11* is located on chromosome 18q21.33. To date, there have been no reports of naturally occurring alterations of clade B serpins leading to a disease phenotype in humans. The only disease association of SERPINB11 is with endometroid ovary carcinoma [28]. Of the six characterized NDNCs that do not have an associated genetic alteration to date, none map to this region, including isolated congenital onychodysplasia (NDNC7), which phenotypically resembles HWSD with thinning and splitting at the distal nail edge of all finger and toenails [8]. Of interest, *SERPINB11* was identified as a potential candidate gene for adaptive evolution in Yoruba [29].

Another mechanistic alternative is that SERPINB11 functions as member of a protein chaperoning complex, similar to the role of SERPINF1 and SERPINH1 in association with procollagen. A loss-of-function alteration in *SERPINF1* has been demonstrated to cause osteogenesis imperfecta type VI in humans [30] while variants in *SERPINH1* have been associated with ostogenesis imperfecta in both humans [31] and dogs [32]. In a similar manner, *SERPINB11* may have a role as a chaperone protein for those proteins involved in hoof wall structure.

### cDNA Sequencing and Tissue Expression of *SERPINB11*

In the horse, we identified one full-length transcript variant from hoof capsule. In humans, there have been seven major *SERPINB11* transcripts identified; three correspond to a protein product with various splice variants and one contains an insertion, leading to a frameshift and premature stop codon at position 90 that results in a nonfunctional variant (NP_001278207) [9]. There are no deleterious effects of this truncated transcript reported in humans. However, based on the results from this study, further investigation into the potential role of the truncated transcript in nail health may be warranted. Tissue expression of *SERPINB11* in humans demonstrates expression in the tonsil, lung, placenta and prostate while *Serpinb11* demonstrates expanded expression in mice with transcripts identified in eye, lung, lymphocytes, thymus, stomach, uterus, heart, brain, liver, skeletal muscle and whole embryonic tissue at day 7, 15 and 17 [9]. Tissue expression of *SERPINB11* in the horse was similar to mouse and included lung, stomach and brain (Fig 4A). However, expression of *SERPINB11* in the horse was strongest in the coronary band, or most proximal part of the hoof capsule. The coronary band is the tissue from which the dorsal hoof wall arises and is analogous to a human cuticle. To the authors’ knowledge, expression of *SERPINB11* in skin and nail tissue has not been examined in humans and mice.

### Conclusions

The results of this study demonstrate a strong association of the *SERPINB11* c.504_505insC variant with the HWSD phenotype. Additionally, HWSD is the first disease to be described that results in a hoof-specific phenotype, with no other ectodermal structures affected. Further studies are necessary to determine the mechanism by which SERPINB11 maintains structural integrity of the hoof wall of healthy ungulates and if SERPINB11 plays a similar role in nail and claw health of non-ungulate species.

## Materials and Methods

### Ethics Statement

Blood samples from index cases were collected at the University of California, Davis School of Veterinary Medicine William R. Pritchard Veterinary Medical Teaching Hospital. Additional samples were drawn by private veterinarians and mailed by individual Connemara owners. All animal samples were obtained following protocol number 17491 approved by the University of California Davis Institutional Animal care and Use Committee.

### Phenotype

Two affected Connemara ponies were evaluated at the University of California, Davis (UCD) Veterinary Medical Teaching Hospital in 2011 by a board-certified equine internist (CF). Following humane euthanasia, hooves from three additional affected ponies were evaluated by a board-certified pathologist (VKA). Distal extremity radiographs were available from the five index cases. From these index cases and the histologic assessment of affected hooves, the phenotype of HWSD was established. For additional cases, inclusion criteria as an HWSD case consisted of (1) Connemara pony breed (2) age of onset within the first six months of life and (3) clinical signs consistent with a receding dorsal hoof wall and secondary solar proliferation, supported by digital photographs of all four feet. Inclusion criteria for unaffected animals in the genome wide association study consisted of (1) Connemara pony breed (2) >2 years of age (3) no apparent hoof pathology, supported by digital photographs of all four feet when available. Distal extremity radiographs of affected horses were evaluated, when available. Control animals used for genotyping of putative functional variants were reported by their owners to be >2 years of age and had no apparent hoof pathology.

### Genomic DNA

DNA was collected and purified from all horses (Gentra Puregene blood kit, Quiagen, Valencia, CA).

### RNA and cDNA

Hooves from four affected Connemara ponies [3 female (aged 2.5, 3, and 4.5 years) and 1 male (6 months)], including three of the index cases, were available for RNA purification. Four unaffected [2 female (1.5 years), 2 male (1 and 5 years)] horses were euthanized for reasons unrelated to this study and hooves collected as controls. All tissue samples were flash frozen in liquid nitrogen and stored at -80^°^C until RNA isolation. RNA was isolated (RNeasy Fibrous Tissue Mini Kit, Quiagen, Valencia, CA) and cDNA synthesized (QuantiTect Reverse Transcription Kit, QIAGEN, Valencia, CA). Negative reverse transcriptase controls were made simultaneously and the final products were assessed with the housekeeping gene, *ACTB*, as previously described [33].

### Genome Wide Association Study

15 affected (4 male, 11 female) and 24 unaffected (7 male, 17 female) Connemara ponies were genotyped on the Illumina SNP70 Genotyping Beadchip (Illumina, San Diego, CA). Quality control was implemented using PLINK (Purcell et al 2007) and Single Nucleotide Polymorphisms (SNPs; based on EquCab2.0) were excluded with a minor allele frequency (MAF) <5% or genotyping call rate <90%. A case-control standard allelic genome-wide association (GWA) was performed using PLINK. Population stratification was determined using genomic inflation values in PLINK and visualized using multi-dimensional scaling (MDS) and quantile-quantile (Q-Q) plots. After removing seven control animals based upon the MDS plot, a repeat case-control standard allelic GWA analysis was performed with the remaining 15 affected and 17 unaffected (4 male, 13 female) animals. To correct for multiple testing, 52,000 permutations were performed. Manhattan plots and QQ plots were generated using the ggplot2 package [34] implemented in R v.3.1.1 [35].

### Whole-Genome Next Generation Sequencing

Sequence data was generated using an Illumina HiSeq. Library preparation and sequencing was performed by the UC Davis Genome Center. Average library insert size was 300 bases. Four samples (2 affected [1 female, 1 male] and 2 unaffected [1 female, 1 male]) were bar-coded and pooled and 2 lanes of sequence of 100bp paired-end reads were obtained. An average of 79.2M reads were obtained per sample. Average read length after trimming was 98.25bp, resulting in 5.4-6X coverage for each of the four horses. Sequence data was processed on a 2U custom built rack server with 256GB DDR3 memory, 2 x Xeon(R) E5-2690 Eight-Core Processor (32 virtual cores), 22TB HD and Ubuntu 12.04.2 operating system. Read quality was assessed using qrqc (version 1.9.1) [36], while Scythe (version 0.990) [37] and Sickle (version 1.20) [38] were used for Illumina adapter & quality trimming. The Burrows-Wheeler Aligner (BWA version 0.6.2-r126) [39] was used to align reads to the horse genome (UCSC assembly ID: equCab2). Sequenced reads from an American Quarter Horse [13] were downloaded and used as an additional control. To call variants, the Genome Analysis Toolkit (GATK version 2.5-2-gf57256b) [40] best practices for variant calling was followed, including duplicate removal, indel realignment, base quality score recalibration, and SNP and INDEL discovery and genotyping across all samples using GATK recommended hard filtering parameters [41,42]. Variant effect prediction was assessed using SnpEff [21] (version 3.5f) [43]. Of the segregating variants identified, UCSC was used to identify the syntenic region on hg19 [12] and and the ECR browser (http://ecrbrowser.dcode.org/) used to identify potential highly conserved non-coding regions. FASTq files have been uploaded for the sequence data to the NCBI-SRA (submission # SRP052751).

### SNP and Indel Genotyping

A custom Sequenom SNP panel (GeneSeek, Lincoln, NE) was designed to genotype the 17 SNPs and 5 indels that segregated with the affected phenotype on an additional 369 Connemara ponies, 18 non-Connemara ponies, 50 Arabians, 51 Quarter Horses, and 50 Thoroughbreds_._ An additional 54 Connemara ponies were genotyped only for *SERPINB11*, using the forward primer CAAGGGGATGAGGGAGTTCT and reverse primer CCTCACTTAGCCGAAAAGGA; the 296bp product was sequenced and assessed for the presence of a 1bp insertion.

### Expression Analysis

RT-PCR was performed to evaluate relative expression of *SERPINB2*, *SERPINB8*, *SERPINB10*, *SERPINB11* in the spleen, heart, lung, kidney, stomach, liver, skin, coronary band, brain, and spinal cord of one control horse. The predicted cDNA sequences for each *SERPIN* gene were pulled from the equCab2 assembly, as viewed on the UCSC Genome Browser [12] and PRIMER3 software [44] was used to design primers (S1 Table). Beta-Actin cDNA was also amplified to ensure equal loading of template, with previously published primers [45]. PCR was performed with the following cycling conditions: 12 minute melt at 95^°^C; 40 cycles of 30 seconds at 94^°^C, 30 seconds at 58^°^C, and 3 minutes at 72^°^C; final 20-minute extension at 72^°^C. PCR products were Sanger sequenced to ensure that the correct product was amplified. The *SERPINB11* primer set was also used to sequence multiple affected ponies to ensure that the frameshift variant was detectable in transcribed cDNA.

### Quantitative Expression Analysis

Primers for the four *SERPIN* genes (*B2*, *B8*, *B10*, *B11*) surrounding disease-associated variants were designed using the PRIMER3 software loaded with server settings for qPCR (Untergasser *et al*., 2012) (S2 Table). cDNA sequences were pulled from the equCab2 assembly [12]. Primers were selected only if pairs spanned at least one intron, did not bind to regions with an identified SNP, produced a single product with In-Silico PCR, and showed a single BLAT search result. Primers were used to PCR amplify from control coronary band cDNA, and products were sequenced to confirm specificity. Three reference genes were identified as potential internal controls: beta-actin (*ACTB*), beta-2-microglobulin (*B2M*), and ubiquitin B (*UBB*) [33]. All primers were synthesized by Eurofins MWG Operon, Huntsville, AL, USA (http://www.operon.com). Housekeeping gene stability was determined by calculating Ct mean, standard deviation, and coefficient of variation [46]. *B2M* demonstrated the most stable expression profile across our eight coronary band cDNA samples. qRT-PCR Reactions were performed in a 10uL reaction volume using the QIAGEN Rotor-Gene SYBR Green PCR Kit. Primers are listed in S2 Table. Each tube contained 20ng cDNA, and 0.5μM final primer concentration for each forward and reverse primer. PCR was performed on a Rotor-Gene Q 72-well thermocycler (QIAGEN, Valencia, CA) as follows: 5 minutes at 95^°^C; 40 cycles of 20 seconds at 95^°^C and 40 seconds at 60^°^C; melt curve ramping from 50^°^C to 99^°^C, rising by 1^°^C at each step. Each reaction was run in triplicate. Each run included a no-template control, negative-RT control, and standard concentration (40ng, 20ng, 10ng, 5ng, 2.5ng) cDNA for each *SERPIN* gene under investigation and *B2M*. Quantitative RT-PCR results were analyzed by group-wise comparison between quantification cycle values obtained from coronary band samples donated by four affected and four unaffected animals. The Relative Expression Software Tool (REST) platform was used to evaluate expression levels [47]. Experiments showing group-based differences were repeated and analyzed in triplicate.

### Carrier Frequency

Pedigree information was collected for all genotyped ponies, using Certificates of Registry issued by regional Connemara societies and the Pedigree Online All Breed Database (www.allbreedpedigree.com). To assess carrier frequency in the general Connemara population, a sample set that excluded affected individuals and close relatives (siblings, half-siblings, sires, dams, grand-sires, and grand-dams) was created. Carrier frequency was calculated by dividing the total number of unrelated animals heterozygous for the frameshift variant by the total number of animals within the unrelated sample set.

## Supporting Information

S1 Table Primers for RT-PCR. (DOCX)

S2 Table Primers for q-RT-PCR. (DOCX)

S3 Table Association results from genome-wide association study sorted by permuted *P* value (EPM2; low to high). CHR = chromosome #, BP = base pair, A1 = allele 1, F_A = Frequency in affected, F_U = Frequency in unaffected, A2 = allele 2, OR = odds ratio, EMP2 = permuted *P* value following 52,000 permutations.
(XLSX)

S4 Table Genotypes of genome-wide significant associated region on chromosome 8 from 15 HWSD-affected (left of the black line) and 17 control ponies (right of black line). Highlighted (yellow) SNPs achieved genome-wide significance. The blue highlighted SNPs denote the region of homozygosity in HWSD-affected ponies.
(XLSX)

S5 Table Variant call format (vcf) file with SNPEff results (Effect) of 363 segregating SNPs from whole-genome sequencing of two HWSD-affected Connemara ponies (CON3, CON26), two control Connemara ponies (CON15, CON40) and one Quarter Horse reference sequence (QH) [13]. (XLSX)

S6 Table Variant call format (vcf) file with SNPEff results (Effect) of 28 segregating indels from whole-genome sequencing of two HWSD-affected Connemara ponies (CON3, CON26), two control Connemara ponies (CON15, CON40) and one Quarter Horse reference sequence (QH) [13]. (XLSX)
