# Supplementary material for: SERPINB11 Frameshift Variant Associated with Novel Hoof Specific Phenotype in Connemara Ponies
Source: PLoS Genet. 2015 Apr 13;11(4):e1005122. doi: 10.1371/journal.pgen.1005122 (PMC4395385; doi:10.1371/journal.pgen.1005122)
Supplement: S1 Table — (DOCX) [file pgen.1005122.s001.docx]

| Gene | Forward Primer | Reverse Primer | Product Length |
| --- | --- | --- | --- |
| *SERPINB2* | GTCTGACAGCACCTCAGAGAA | GGCCAGTTCTCCCTGACATA | 1184bp |
| *SERPINB8* | CTCTTCTGTCCTGGCCATGG | CTCTTCTGTCCTGGCCATGG | 878bp |
| *SERPINB10* | AAGGAATCTGGGAACAGCAA | AACAGCTGATGAGGCTACCG | 1529bp |
| *SERPINB11* | CCCTCAGCAGAGCAAATGTT | TGCCACAGAAGAGAATCGTG | 1155bp |
