# Supplementary material for: SERPINB11 Frameshift Variant Associated with Novel Hoof Specific Phenotype in Connemara Ponies
Source: PLoS Genet. 2015 Apr 13;11(4):e1005122. doi: 10.1371/journal.pgen.1005122 (PMC4395385; doi:10.1371/journal.pgen.1005122)
Supplement: S2 Table — (DOCX) [file pgen.1005122.s002.docx]

| Gene | Forward Primer | Reverse Primer | Product Length | Source |
| --- | --- | --- | --- | --- |
| *SERPINB2* | AGCATGTTCCTGTTGCTTCC | TTGTGTCTTTGCTGGTCCAC | 112bp | Primer3 |
| *SERPINB8* | TTAACAAAGCCGGCACTCAG | TTCGGTGTCTTCAGCAAAGG | 149bp | Primer3 |
| *SERPINB10* | GTCCAAGACACCACAGAAAAGC | GGCCTATGACTTGTGGGTTTTC | 121bp | Primer3 |
| *SERPINB11* | GACAAAGGCAATGGCGTTCC | TTCCACCCAAGCGTTAATCG | 130bp | Primer3 |
| *ACTB* | CCAGCACGATGAAGATCAAG | GTGGACAATGAGGCCAGAAT | 88bp | Bogaert, 2006 |
| *B2M* | GTTCCATCCGCCTGGAGATT | GGGGGTCTTTGAGAGTAGAGTG | 182bp | Bogaert, 2006 |
| *UBB* | GCAAGACCATCACCCTGGA | CTAACAGCCACCCCTGAGAC | 206bp | Bogaert, 2006 |
